# Comparative efficacy of Bacillus probiotics and formalin-killed bacterin against *Vibrio anguillarum* in European eel elvers

**DOI:** 10.1038/s41598-026-35298-8

**Published:** 2026-02-02

**Authors:** Hany M.R. Abdel-Latif, Nashwa Abdel-Razek, Riad H. Khalil

**Affiliations:** 1 Department of Poultry and Fish Diseases, Faculty of Veterinary Medicine, Alexandria University, Alexandria, 22758 Egypt; 2 Department of Fish Health and Management, Central Laboratory for Aquaculture Research, Agricultural Research Center, Abbassa, Abo-Hammad, Sharqia, 44662 Egypt

**Keywords:** Probiotics, Vaccines, Vibriosis, Eels, Immunity, Challenge, Immunology, Microbiology, Zoology

## Abstract

This study evaluated and compared the effectiveness of *Bacillus* species probiotics and formalin-killed bacterin to control vibriosis caused by *Vibrio anguillarium* in European eel (*Anguilla anguilla*) elvers. Fish were allocated into four triplicate groups defined as follows: a control non-vaccinated (CON) group that fed on a basal diet without additives, two groups fed on diets supplied with *Bacillus* species probiotics (PRO; Sanolife PRO-F) in a dose of 0.5 g /kg diet (PRO-F1) and 1.0 g /kg diet (PRO-F2), and the fourth group was vaccinated with immersion formalin-killed bacterin and got a booster vaccination at 14th day (VACC). The experiment lasted for 28 days. In the end, the immune responses, including antibody titers, lysozyme activities (in serum and skin mucous), and bactericidal activity, were evaluated. The relative percent survival (RPS) after intraperitoneal challenge with *V. anguillarium* was calculated after an additional 10-day observation period. A one-way ANOVA followed by Dunnett’s multiple comparison test was used to compare the experimental groups (VACC, PRO-F1, and PRO-F2) with the CON group. The results revealed significantly higher antibody titers, lysozyme activities (in serum and skin mucous), and serum bactericidal activity in the VACC and PRO-treated groups over the CON group. Of interest, the lysozyme activities (in serum and skin mucous) and antibody titers were significantly higher in the VACC group over both PRO groups. After the experimental infection, it was noticed that both VACC and PRO significantly protected eel elvers against *V. anguillarium* compared to the CON group. In addition, the RPS were 41.67%, 29.17%, and 37.50% in the VACC, PRO-F1, and PRO-F2 groups, respectively. This study unveils the vital roles of PRO and VACC and their effective roles in enhancing innate and mucosal immunities and protecting European eel elvers against vibriosis caused by *V. anguillarium*. Based on our findings, immersion vaccination emerges as the superior strategy for managing vibriosis in eels. This approach offers greater protection than feed-based probiotics, underscoring its potential as a viable vaccination method in eel aquaculture.

## Introduction

European eel, *Anguilla anguilla* (Linnaeus, 1758) is an important and highly economic fish species in most European and North African countries^1^. Among the most common anguilliculture diseases, vibriosis is from bacterial diseases that constitute a major problem and severe economic loss in farmed eels^2,3^. In eels, vibriosis is caused by a variety of vibrio species. However, *Vibrio anguillarum*^4,5^, *V. vulnificus* serovar E^6^, and *V. vulnificus* serovar A^7^ are the most common vibrio species isolated from eels. *Listonella anguillarum* (formerly known as *V. anguillarum*), is a Gram-negative, rod-shaped, motile by polar flagella, a non-spore-forming, halophilic and facultatively anaerobic bacterium^8^. It was first discovered in European eels and caused a disease known as the “red pest of eels”^9^. This bacterium is also the causative agent of a devasting and deadly hemorrhagic septicemic bacterial disease affecting a range of marine and fresh/brackish water fish, bivalves, and crustaceans^10^. This disease is responsible for significant mortalities and high economic loss in several countries worldwide^11,12^.

Antibiotics are repeatedly used in aquaculture for the treatment and control of bacterial diseases affecting fish such as vibriosis^13^. However, their application in aquaculture and food animals has been banned in several countries throughout the globe for several reasons such as their hazardous outcomes on human consumers^14^, the emergence of antibiotic-resistant strains^15^, and environmental risks and potential hazards^16,17^. For these reasons, researchers and aquaculture scientists have paid considerable attention to finding effective and environmentally safe substitutes instead of antibiotics.

Vaccination is a promising biosecurity strategy to improve immune responses and confer significant protection for fish against a wide range of challenging pathogens^18^. Several vaccines have been developed for fish vaccination with proven efficacy and noticeable protection against many fish diseases^19,20^. An earlier trial was carried out by Fouz et al.^21^, who reported a successful field vaccination of European eels (glass stage) against *V. vulnificus* serovar E with noticeable survival rates of the vaccinated eels. Further, prolonged immersion vaccination using “Vulnivaccine” conferred considerable protection against *V. vulnificus* serovar E in glass eel stage^22^ and market-sized eels^23^. Further research elucidated that a bivalent vaccine against *V. vulnificus* serovar A and serovar E has been developed and successfully provided protection for eels against vibriosis caused by both serovars^24^.

Probiotics are feed or water-based microbial supplements that can reach the gastrointestinal (GI) tract of fish and beneficially maintain the intestinal microbial balance, enhance the immune responses, disease resistance, health status, growth performance, and modulate stress responses^25–28^. In eels, it was reported that dietary supplementation with a probiotic strain, *Lactobacillus pentosus* PL11 enhanced the immunity and resistance of the Japanese eel (*Anguilla japonica*) against *Edwardsiella tarda* infection^29,30^. Dietary supplementation with *Lactobacillus plantarum* KCTC3928 significantly boosted the immune responses and resistance of *A. japonica* against *V. anguillarum* infection^31^. Moreover, it was found that the combined dietary application of *Bacillus subtilis* WB60 and mannan oligosaccharides enhanced the growth, intestinal histology, and resistance of *A. japonica* against *V. anguillarum*^32^. A recent study demonstrated that *Bacillus* sp. FEB-1 probiotic supplementation significantly improved the survival rates of *A. japonica* larvae^33^.

No previous studies have compared the effectiveness and efficacy of probiotics and vaccination in terms of innate and mucosal immunities and resistance to *V. anguillarum* infection in European eels. Therefore, this research study aimed to investigate the effects of oral administration of *Bacillus* species probiotics or the use of FKC bacterin on the innate and mucosal immunities of eel elvers. Additionally, their effectiveness in protecting eels against vibriosis caused by *V. anguillarum* infection was examined. The findings of this study may pave the way for identifying optimal biosecurity strategies to control vibriosis in eels and enhance their aquaculture productivity.

## Materials and methods

### Fish rearing, acclimation and husbandry

A total of 120 healthy European eel (*Anguilla anguilla*) elvers (approx. 25–30 g body weight) were reared in tanks located at Agromar Company for Agriculture Investment, Cairo-Alexandria Desert Road, Egypt. For acclimation, fish were maintained in equal-sized 500-L plastic tanks at 27 ± 2 °C with a 24-h dark period for 2 weeks prior to the experiment. They were fed 3% of their body weight twice a day, with an antibiotic-free commercial diet (DIBAQ DIPROTEG, Spain). Its proximate chemical composition is crude protein 45%, crude lipids 19.5%, crude fiber 1.1%, ash 11%, N-free extract (NFE) 23.4, and gross energy (GE) 22.6 MJ/kg).

### Water quality parameters

The physical and chemical parameters of the rearing water were analyzed on-site during farm visits. A multi-parameter electrochemical analyzer (model C6030 Consort, Belgium) was used to measure water temperature, pH, dissolved oxygen (DO), and salinity. Total ammonia, nitrite, total hardness, and alkalinity levels were determined using an HI83399 photometer (HANNA Instruments, Italy) with the following test kits: ammonia low range (HI93700-1), nitrite low range (HI93707-01), total hardness low range (HI93735-00), and alkalinity (HI755-26). These measurements were performed according to the reference APHA methods. The unionized (toxic) ammonia levels were calculated using the online free ammonia-nitrogen calculator. The mean values of salinity ((‰, DO (mg/L), total soluble solids (mg/L), total hardness (mg/L), pH value, alkalinity, hydrogen sulfide (mg/L), unionized ammonia (mg/L), nitrate (mg/L), and nitrite (mg/L) were 21.0 ± 1.5, 5.6 ± 0.5, 33.2 ± 0.5, 13.3 ± 0.2, 7.8 ± 0.2, 138.8 ± 1.2, 3.5 ± 0.2, 0.02 ± 0.1, 1.6 ± 0.1, and 0.07 ± 0.01, respectively.

### Experimental setup

**Fig. 1:**
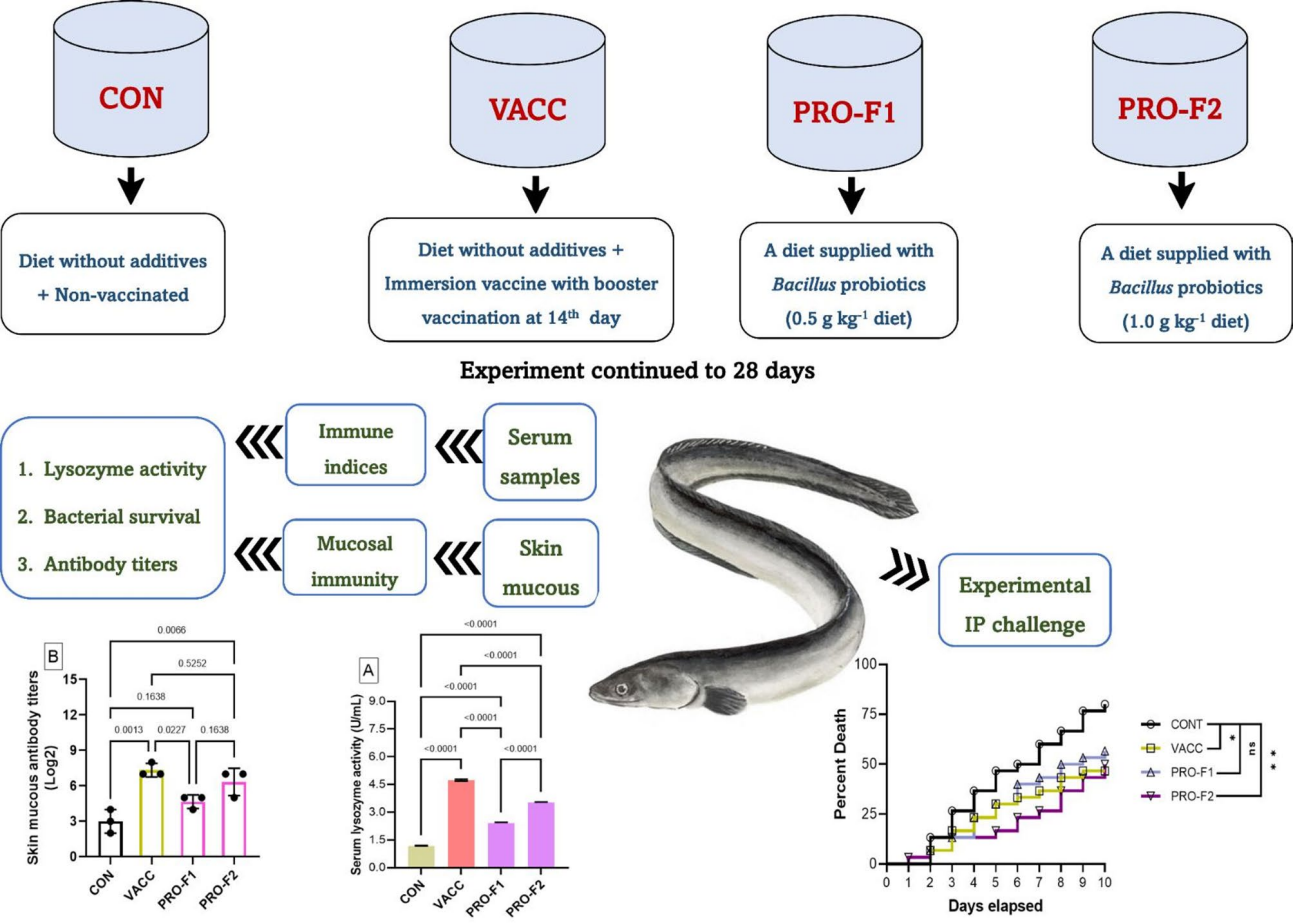
A schematic diagram showing the experimental design of the present study.

Eel elvers were distributed into triplicate groups (each replicate contains 10 fish). Fish were raised in equal-sized tanks which were supplied with continuous aeration. The experiment continued for a continuous 28 days. Figure 1 showed a schematic diagram of the experimental design of the present study. Fish groups were defined as control group (CON), vaccinated (VAC), and two probiotic supplemented groups defined as PRO-F1 and PRO-F2. The **CON** is a control group that was fed on a commercially purchased diet without any supplement. The **VACC** is a vaccinated group which fed on a commercially purchased diet without any supplement and was vaccinated with an immersion whole cell formalin-killed bacterin prepared from *V. anguillarum* strain that was kindly provided from the Department of Poultry and Fish Diseases, Alexandria University^34^. The method used for vaccine preparation has been described in detail in our recently published research^19^. In brief, for bacterial inactivation, the cultures were suspended in a 3% formalin along with nonstop agitation at 25 °C for 24 h. The prepared solution was centrifuged at 1800 × g for 30 min, whereas supernatant was discarded, and the bacterial pellets were collected and then re-suspended 3 times in phosphate-buffered saline (PBS; pH 7.4). For safety testing, 100 µL of the prepared formalin-inactivated material was plated on the tryptic soy agar (TSA) medium and then incubating at 30 °C for 3 days to observe any bacterial growth. This group received a booster vaccination at 14th day after the first dose. Finally, **two probiotic supplemented groups** were fed on diets supplied with *Bacillus* species probiotics (PRO; Sanolife PRO-F, INVE Aquaculture, Belgium). This commercial product is composed of (a combination of *Bacillus subtilis* 3.25 × 10^9^ CFU/g, *Bacillus licheniformis* 3.50 × 10^9^ CFU/g, and *Bacillus pumilus* 3.25 × 10^9^ CFU/g). This product was admixed with the commercial diets using sunflower oil (20 ml/kg diet). Two probiotic doses were used in a dose of 0.5 g/kg diet (**PRO-F1**) and 1.0 g/kg diet (**PRO-F2**). These doses were selected according to the recommendation provided by El-Son et al.^35^.

### Serum and skin mucus collection

At the end of the experiment, eels were fasted for 48 h prior to sampling. Sera and skin mucous samples were collected from 9 elvers per group (pooled into 3 samples; *n* = 3). Blood samples were collected first and were left in the collecting tubes in a standing position overnight to separate serum. Sera samples were obtained from anesthetized elvers according to the method described by Collado et al.^36^. Non-anesthetized elvers were placed individually in empty and sterile flasks. Mucus sampling mucous was performed immediately after the eel is removed from the water by gentle scraping the skin with a sterile glass coverslip along the dorsal surface or lateral line of the sampled eels to avoid contamination from the intestinal or urinogenital secretions. The collected mucus is transferred into small 1.5mL sterile tubes for subsequent processing. The mucus was then filtered sequentially through 0.8 μm and 0.45 μm pore-size membranes. Mucus samples were used immediately, while serum samples were stored at −80 °C until further analysis, following the method illustrated by Esteve-Gassent et al.^37^.

### Immunological measurements

#### Lysozyme activity

Lysozyme activity in sera and skin mucous samples was determined using a lyso-plate assay, as described by Esteve-Gassent et al.^22^. This assay is based on the lysis of *Micrococcus lysodeikticus*. Chicken egg white lysozyme at varying concentrations was used as a standard. The Lysozyme activity unit was defined as the amount of enzyme yielding a decrease in absorbance of 0.001/min.

#### Bacterial survival

The effects of sera and skin mucous samples of elvers on the growth and survival of *V. anguillarum* were measured according to the method described by Amaro et al.^38^ and Esteve-Gassent et al.^23^. In brief, a bacterial suspension of 10^5^ CFU/mL in PBS was mixed with each test sample. The mixtures were incubated at room temperature, and samples were taken at 0 and 3 h for bacterial counts on Trypticase Soy Agar (TSA; Oxoid, Germany) supplemented with 0.5% NaCl.

#### Quantification of the antibody levels

The antibody titers were quantified using the microtiter plate agglutination test^39,40^. In short, 96-well microtiter plates with rounded bottoms were plated with 25 µL of PBS. Afterward, 25 µL of the sample (eel serum or mucous) was added in each well of the first row and then mixed well. Next, twofold serial dilutions were performed. Then, 50 µL of the bacterial cell suspension (9 × 10^8^CFU/mL) was added and mixed with the content in each well. Plates were covered and incubated overnight at 28 °C and then for 4 h at 4 °C before reading. These steps were carried out in agreement with the technique depicted by Shoemaker et al^41^. The agglutination endpoint was visually observed as the final dilution, whereas visible agglutination was seen and was chosen as the agglutinating antibody titer. The titers were expressed as Log2 (x + 1) of the mutual of the highest dilution of the test samples that exhibited obvious agglutination compared to the positive control as detailed in our recently published research^19^.

### Bacterial challenge test

The concentration of the bacterial strain that used for the challenge test was determined by using the pour plate count method as previously described in Harrigan and McCance^42^, whereas a bacterial inoculum of 0.2 mL bacterial suspension (4.67 × 10^7^ colony-forming units (CFU)/fish) of virulent strain of *V. anguillarum* that used for vaccine preparation. This bacterial dose was selected based on the LD50 test that was performed prior to the experimentation (data was not shown). All fish in all groups were IP injected. The specificity of death was verified after re-isolation of the bacterial pathogen used for the challenge test from freshly dead fish for 10 days (post challenge observation period), mortalities were daily monitored, and the protection level was evaluated after challenge by calculating the relative percentage of survival (RPS)^43^. The mortality rate between test groups was compared by the Kaplan-Meyer survival method, followed by the Log-rank test at *P < 0.05 and **P < 0.01.

### Statistical analysis

Data were expressed as means ± S.E. The comparison between the treatment was performed by Dunnett’s multiple comparison test with significance levels at *P* < 0.05, *P* < 0.01, and *P* < 0.0001. Data were analyzed using SPSS statistics software (SPSS Inc., Chicago, IL, USA) and GraphPad prism X8 program.

## Results

### Lysozyme activity

**Fig. 2:**
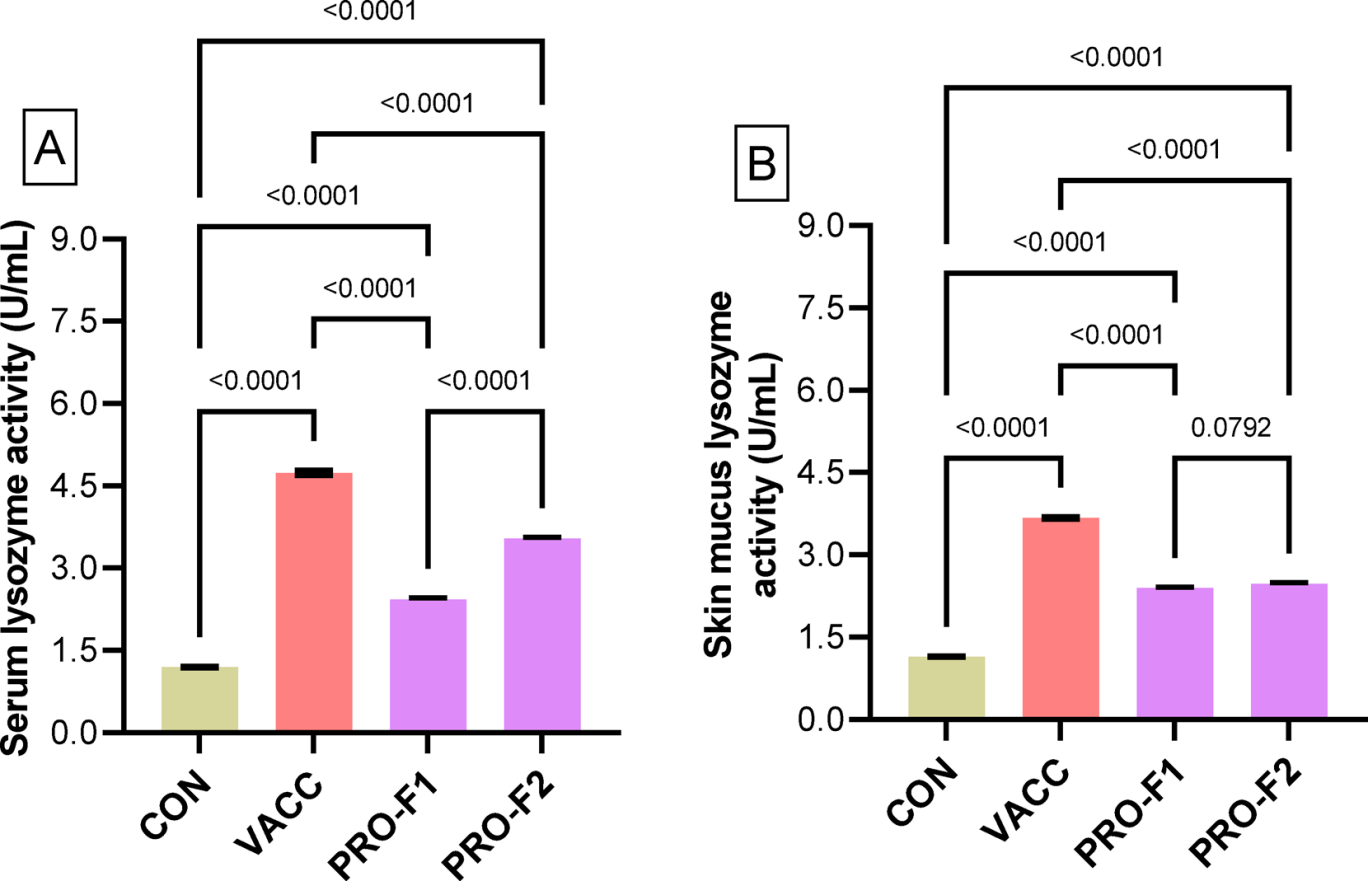
Lysozyme activity (Units/mL) in serum (**A**) and skin mucus (**B**) obtained from European eel elvers that were immunized with immersion FCK bacterin (**VACC**) or those fed on diets supplied with *Bacillus* species probiotics (0.5 g/kg diet; **PRO-F1**) or (1 g/kg diet; **PRO-F2**), and control non-vaccinated group that fed on a basal diet (**CON**). Samples were taken on the 28th day of the experiment. Data were expressed as means ± SE of three biological replicates and three technical replicates per group (*n* = 3). The comparison between the experimental groups (**VACC**, **PRO-F1**, and **PRO-F2**) with the control (**CON**) group was conducted by one-way ANOVA analysis followed by Dunnett’s multiple comparison test.

The lysozyme activity in serum (Fig. 2A) and skin mucus (Fig. 2B) of eel elvers were significantly higher (*P* < 0.05) in the VACC, PRO-F1, and PRO-F2 groups compared to the CON group. Moreover, their values in the VACC group were significantly higher (*P* < 0.05) than in the PRO groups.

### Bactericidal activity

**Fig. 3:**
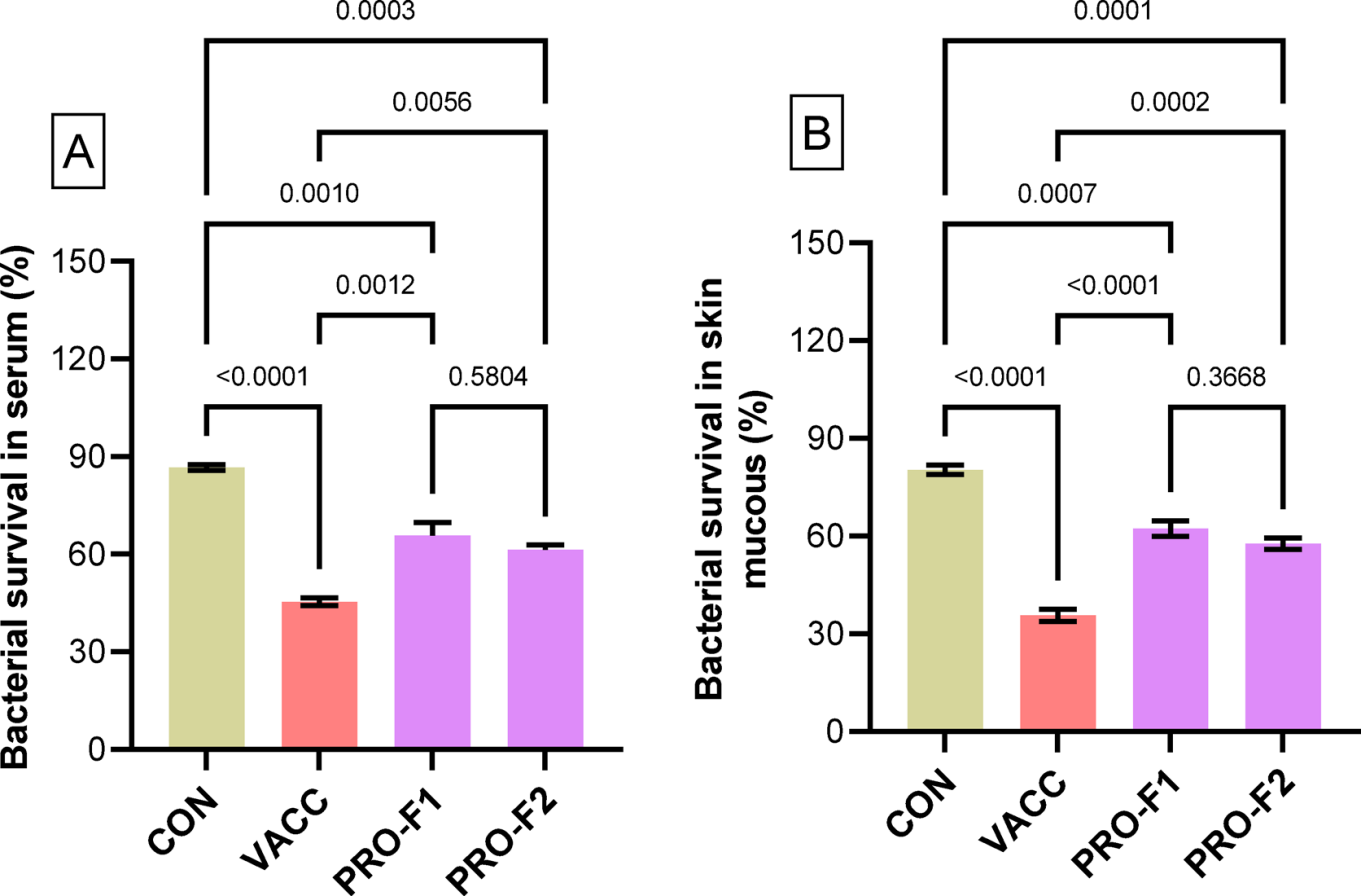
Survival (%) of *V. anguillarium* strain after 3 h incubation in serum (**A**) and skin mucus (**B**) obtained from European eel elvers that were immunized with immersion FCK bacterin (**VACC**) or those fed on diets supplied with *Bacillus* species probiotics (0.5 g/kg diet; **PRO-F1**) or (1 g/kg diet; **PRO-F2**), and control non-vaccinated group that fed on a basal diet (**CON**). Samples were taken on the 28th day of the experiment. Data were expressed as means ± SE of three biological replicates and three technical replicates per group (*n* = 3). The comparison between the experimental groups (**VACC**, **PRO-F1**, and **PRO-F2**) with the control (**CON**) group was conducted by one-way ANOVA analysis followed by Dunnett’s multiple comparison test.

The bactericidal activity in serum (Fig. 3A) and skin mucus (Fig. 3B) of eel elvers revealed significantly higher (*P* < 0.05) levels in the VACC, PRO-F1, and PRO-F2 groups compared to the CON group. Moreover, the higher survival (%) of *V. anguillarium* after 3 h incubation was observed in the CON group, and the lowest survival (%) was noticed in the VACC group. Of interest, the bacterial survival (%) was significantly unaffected (*P* > 0.05) between the PRO-F1 and PRO-F2 groups.

### Serum antibody titers

**Fig. 4:**
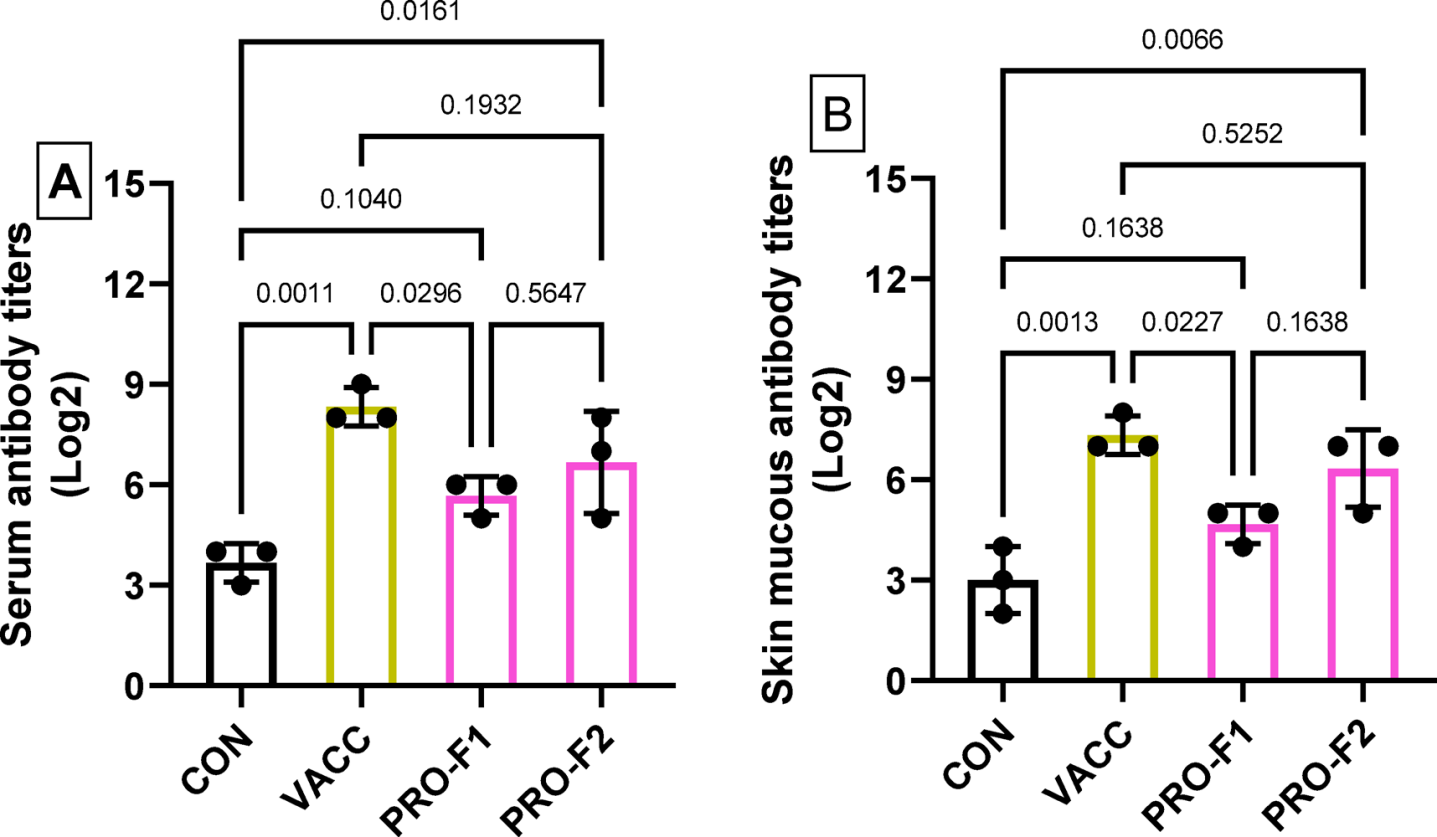
Antibody titers in serum (**A**) and skin mucus (**B**) obtained from European eel elvers that were immunized with immersion FCK bacterin (**VACC**) or those fed on diets supplied with *Bacillus* species probiotics (0.5 g/kg diet; **PRO-F1**) or (1 g/kg diet; **PRO-F2**), and control non-vaccinated group that fed on a basal diet (**CON**). Samples were taken on the 28th day of the experiment. Antibody titters were determined by the Microtiter plate agglutination test. Data were expressed as means ± SE of three biological replicates and three technical replicates per group (*n* = 3). The comparison between the experimental groups (**VACC**, **PRO-F1**, and **PRO-F2**) with the control (**CON**) group was conducted by one-way ANOVA analysis followed by Dunnett’s multiple comparison test.

The antibody titers in serum (Fig. 4A) and skin mucus (Fig. 4B) of eel elvers were significantly elevated (*P* < 0.05) in the VACC, PRO-F1, and PRO-F2 groups than in the CON group. Serum and mucous antibody titers in the VACC group were significantly (*P* < 0.05) higher than the CON and PRO groups. Antibody titers (in serum and skin mucous) were statistically similar (*P* > 0.05) in the PRO-F1 and PRO-F2 groups.

### RPS and experimental infection results

**Fig. 5:**
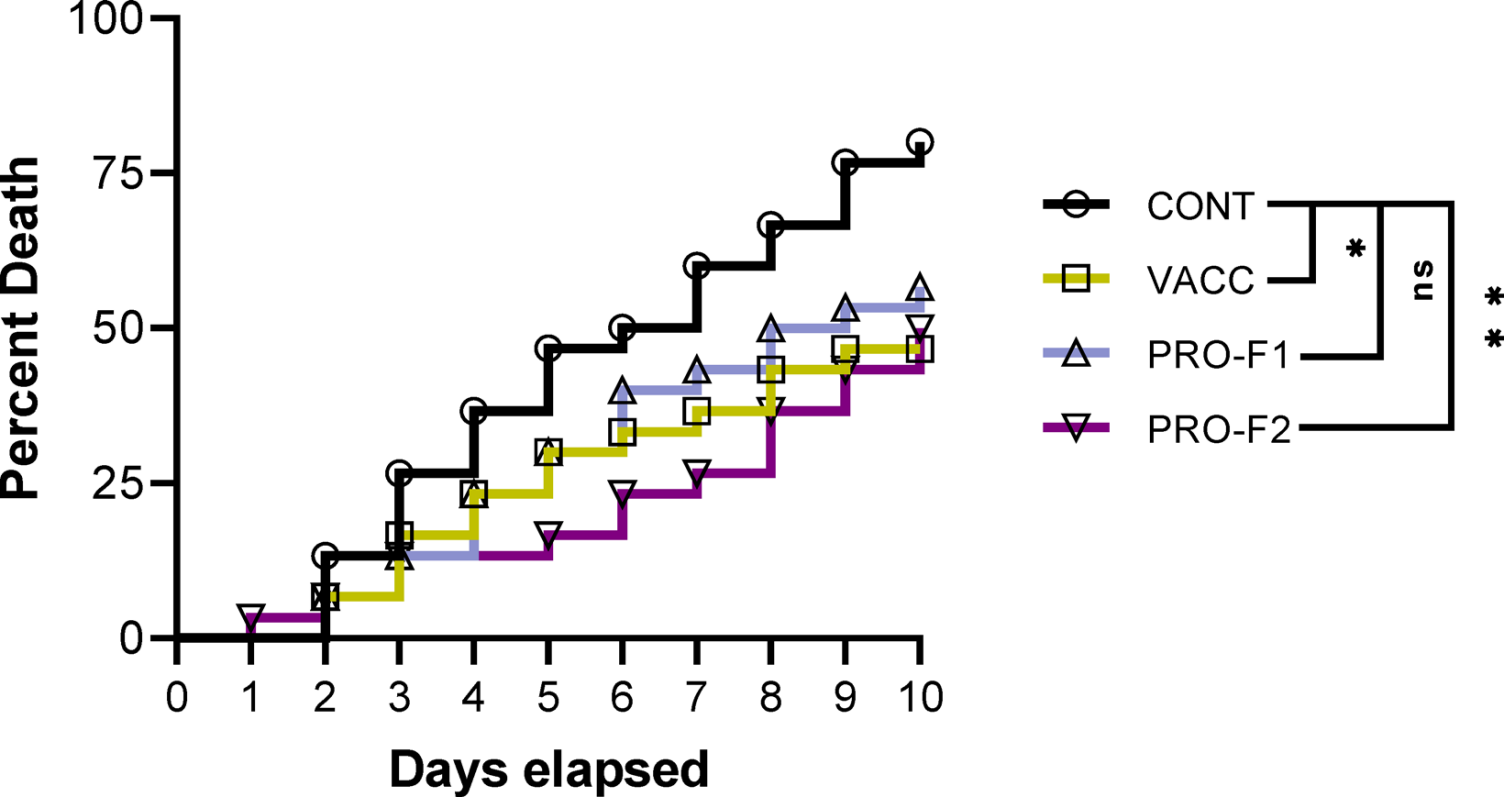
Cumulative mortality of European eel elvers that were immunized with immersion FCK bacterin (**VACC**) or those fed on diets supplied with *Bacillus* species probiotics (0.5 g/kg diet; **PRO-F1**) or (1 g/kg diet; **PRO-F2**), and control non-vaccinated group that fed on a basal diet (**CON**). The IP challenge test was performed, and mortalities were recorded for 10 days post-challenge with *V. anguillarium*. Asterisks denote significant differences (*) (*P* < 0.05) and (**) (*P* < 0.01) between experimental groups, as determined by the Kaplan-Meyer survival method followed by the Log-rank test. Ns means not significant.

The post-challenge clinical signs demonstrated the typical signs of eel vibriosis caused by *V. anguillarium* and characterized by dermal hemorrhages especially at the base of fins and around the vent, skin ulcers, corneal opacity and distended abdomen. According to Fig. 5, the mortality in the PRO-F2 group started on the first day post-challenge while the mortality in the CON, VACC, and PRO-F1 groups started on the second day. Mortality continued in all test groups up to 10 post-day challenges. The Kaplan-Meyer survival method followed by the Log-rank test (Fig. 5) revealed that the survival rate significantly improved the VACC and PRO groups compared to the survival rate in fish fed the CNT diet, suggesting that both immersion vaccination and feed-based probiotics significantly protected eel elvers against *V. anguillarium* compared to the CON group. However, there was no significant difference between the PRO-F1 and VACC groups. Moreover, the RPS (Table 1) were 41.67%, 29.17%, and 37.50% in the VACC, PRO-F1, and PRO-F2 groups, respectively.

**Table 1:** Mortality rate (MR; %), survival rate (SR; %), and relative percent survival (RPS) of European eel Elvers that were immunized with immersion FCK bacterin (**VACC**) or those fed on diets supplied with *Bacillus* species probiotics (0.5 g/kg diet; **PRO-F1**) or (1 g/kg diet; **PRO-F2**), and control non-vaccinated group that fed on a basal diet (**CON**) for 28 days and then experimentally challenged with *V. anguillarium* and observed for 10 days.

Experimental groups	No. of challenged eels	MR (%) ^1^	SR (%)^1^	RPS ^2^
**CON**	30	80.00 ^a^	20.00 ^d^	-
**VACC**	30	46.67 ^c^	53.33 ^a^	41.67
**PRO-F1**	30	56.67 ^b^	43.33 ^c^	29.17
**PRO-F2**	30	50.00 ^b^	50.00 ^b^	37.50

^1^ Values with different superscript letters in the same column are significantly different within groups (*P* < 0.05).

* MR (%) = [No. of dead eels/Total No. of challenged eels] × 100.

^#^ RPS (%) = [1 – (Mortalities of the test group/Mortalities of **CON** group)] × 100.

## Discussion

The immune system of eels comprises a complex immune system as it includes both innate and adaptive components^44^. The innate immune system represents the first line of defense mechanisms, depending on many factors such as antimicrobial peptides and proteins (in serum and skin mucous) which help in the inhibition of pathogen colonization in the mucosa, and cellular responses (like macrophages and neutrophils) (which is involved in phagocytosis which helps to engulf and destroy the challenging pathogens)^45^. Compared to mammals, the adaptive immune system of eels is less developed and involves lymphocytes and antibodies production (especially the mucosal immunity in gills, guts, and skin), the presence of mucosal-associated lymphoid tissues (MALT) (in the gills, gut, and skin) plays a crucial role in the immune response to pathogens entering through these surfaces^46^.

Vaccination in anguilliculture has been greatly developed with the aim of improving the immune responses and conferring considerable protection for fish against a wide range of challenging pathogens, especially Vibrio species^21–24^. On the other hand, the trend of using probiotics in controlling vibriosis in fish has been flourishing in the current days because of their potential to enhance immunity and provide a disease control strategy in aquaculture^11,13^. *Bacillus* spp. probiotics are abundant in the intestinal tract of fish and are known to provide various beneficial effects to their host^47^. They enhance immunity responses and produce natural antibacterial compounds that help in the antagonization of the challenging pathogens, thereby improving the fish ability to resist diseases^48,49^. The present study investigated the potential of oral administration of *Bacillus* species probiotics or the use of FKC bacterin on the innate and mucosal immunities of eel elvers. Their effectiveness in protecting eels elvers against vibriosis caused by *V. anguillarum* infection was examined.

The results of the present study revealed significantly higher antibody titers, lysozyme activities (in serum and skin mucous), and serum bactericidal activity in the vaccinated (VACC) and probiotic (PRO)-treated groups over the control (CON) group which fed the commercial diet without supplements. Moreover, lysozyme activities (in serum and skin mucous) and antibody titers were significantly higher in the VACC group over both PRO groups. In eels, lysozyme presents an important part of the innate immune system and exhibits its highest activity during the early stages of development^44^. Besides its damaging activity against gram-positive (G + ve) bacterial cell walls, it has antibacterial activity against gram-negative (G-ve) bacteria in the absence of a compliment as it activates phagocytosis^50^. A wide range of research studies unveiled the role of immunostimulants in the increase of lysozyme activity in fish as eels. For example, Lee et al^31^. showed that lysozyme activity was peaked in the Japanese eels that fed diet supplied with 10^8^ CFU/g *B. subtilis*. Lysozyme activity was also increased in Nile tilapia groups fed diets supplemented with *Bacillus* sp. probiotics and vaccinated with ME-VAC Aqua Strept^®^ polyvalent vaccine, as reported by Aboleila et al.^51^. There are several other research studies that demonstrate the roles of *Bacillus* sp. probiotics in increasing the lysozyme activity in fish^35,52,53^. On the other hand, Esteve-Gassent et al.^23^ found that reimmunization of Vulnivaccine mixed with food increased the lysozyme activity in farmed European eels. Several types of vaccines can also increase the lysozyme activity in serum, skin mucus, liver and kidney of American eel^54^ and in Japanese eel^55^.

Fish serum and skin mucus contain several immune molecules such as antibodies, complement, etc. and they act as lines of defense against pathogenic organisms^56^. Serum immunoglobulins (Igs) are major components of the humoral immune system and IgM is the main type of Igs that are present in fish^57^. There are specific Igs that are implicated in bacteria opsonization, toxin neutralization and are potent complement activators^58^. In align with our study, it was found that dietary supplementation of *L. pentosus* significantly increased the plasma immunoglobulin M levels in Japanese eels against *E. tarda*^30^. This probiotic strain also exhibited in vitro competitive exclusion (88.4% reduction) in adhesion to *E. tarda* to the intestinal mucus^59^. Serum immunoglobulin M levels were also significantly increased in Nile tilapia that fed on *Bacillus* sp. probiotic mixture^60^. The antibody titers against *A. hydrophila* and *V. vulnificus* were increased in eels vaccinated by FKC and outer membrane protein (OMP) bivalent vaccines^54^. Higher antibody titers were found in eels vaccinated with different vaccine types^22,36,37^, suggesting the important roles of vaccination in conferring protection against bacterial pathogens. Of interest, the bactericidal effects detected in VACC and PRO-treated fish might be associated with the high specific antibodies titers against *V. anguillarum*^21^.

In the present study both VACC and PRO significantly protected eel elvers against *V. anguillarium* compared to the CON group. In the same sense, it was found that dietary L. pentosus PL11 enhanced the growth performance and the immunity of Japanese eel, resulting in increased resistance to *E. tarda* infection^30^. Dietary *B. subtilis* and *L. plantarum* significantly increased the survival rates of Japanese eels challenged with *V. anguillarum*^31^ due to their positive roles in enhancing the growth performance, humoral immune responses, gut histomorphology of the treated eels. Moreover, a dietary supplementation of *B. subtilis* at 0.5 × 10^7^ CFU/g diet and mannanoligosaccharide at 5 g/kg diet produced beneficial synergistic effects in the increase the resistance of Japanese eel to *V. anguillarum* infection^32^. In another fish species, Abdel-Latif et al^61^. found that a dietary supplementation with a multispecies probiotic enhanced the immunity, and resistance of *Pangasianodon hypophthalmus* fingerlings challenged with *A. hydrophila*. Probiotics can also activate the immune responses of fish^62^. The immunostimulatory effects of *Bacillus* sp. probiotics may be linked to the release of various cytokines that have antimicrobial effects against the pathogenic microorganisms^63^. Probiotics produce several functional substances in the fish intestine (such as bacteriocin, siderophore, and lysozyme), improving the local immune responses in the gut^64^. In addition, probiotics can positively influence host health, primarily by regulating intestinal flora and boosting immunity through the intestinal flora-immune axis. They can enhance the immune system through several mechanisms: strengthening the epithelial barrier, which prevents harmful substances from entering the bloodstream; blocking pathogens from attaching to the intestinal lining; and fine-tuning the maturation and response of the immune system itself. Therefore, they contribute positively to overall host immunity by modulating the gut microbiome in ways that help treat or prevent various diseases^65^.

Vaccines also provide significant protection of eels against bacterial pathogens. For instance, r-OmpK vaccine increased the survival of European eels against the challenge against *A. hydrophila*^66^. The RPS of American eels vaccinated with FKC and OMP vaccines was significantly increased after being challenged with *V. vulnificus*^54^. Vulnivaccine also produced higher survival rates of glass eels challenged with *V. vulnificus* serovar E^21^. The circulating levels antibodies have been believed to be the principal protective immune response against extracellular challenged bacteria^19^. In general, our study demonstrated that VACC provides higher immunity and protection of eels than PRO treatments. These results differed that those reported by Aboleila et al^51^., who found that PRO might present more effective protective roles than VACC for providing effective biological and alternative control strategy in Nile tilapia and can prevent the disease occurrence and maintain optimal health with the potential to overcome the negative effects and drawbacks of using vaccines. This discrepancy might be related to fish species differences and types of vaccines used. However, those authors demonstrated that combining *Bacillus* sp. Probiotics and IP administration of ME-VAC Aqua Strept^®^ polyvalent vaccine provided excellent immune responses, expression of immune-related genes and conferred protection of Nile tilapia against *Streptococcus iniae*^51^.

## Conclusions

The current research demonstrates that both immersion vaccination with FKC bacterin and oral administration of *Bacillus* species probiotics significantly enhance the innate and mucosal immunity of farmed eel elvers. Both strategies effectively protect eel elvers against vibriosis caused by *V. anguillarum* infection. However, the immune responses and protective effects were more pronounced in the vaccinated group compared to the probiotic-supplemented group. Therefore, we recommend immersion vaccination with FKC bacterin as a reliable biosecurity measure for controlling vibriosis in eel farming. While both strategies show promise, further research is needed to evaluate the combined efficacy of immersion vaccination and oral probiotic administration in enhancing immunity and disease resistance in farmed eels. This combined approach may offer the most effective strategy for preventing vibriosis in eel aquaculture. We acknowledge that future research should integrate these biological findings with economic modeling to provide a comprehensive decision-making tool for aquaculture farmers.

## Data Availability

The datasets used and/or analysed during the current study available from the corresponding author on reasonable request.
